# Identifying priority conservation landscapes and actions for the Critically Endangered Javan leopard in Indonesia: Conserving the last large carnivore in Java Island

**DOI:** 10.1371/journal.pone.0198369

**Published:** 2018-06-27

**Authors:** Hariyo Tabah Wibisono, Hariyawan Agung Wahyudi, Erwin Wilianto, Irene Margareth Romaria Pinondang, Mahendra Primajati, Darmawan Liswanto, Matthew Linkie

**Affiliations:** 1 Department of Entomology and Wildlife Ecology, College of Agriculture and Natural Resources, University of Delaware, Newark, Delaware, United States of America; 2 Population Sustainability, San Diego Zoo Institute for Conservation Research, Escondido, California, United States of America; 3 Forum HarimauKita, Bogor, West Java, Indonesia; 4 Biodiversity Society, Banyumas, Central Java, Indonesia; 5 Fauna & Flora International – Indonesia Programme, Pondok Labu, Jakarta, Indonesia; 6 Yayasan Titian Lestari, Pontianak, Kalimantan Barat, Indonesia; 7 Wildlife Conservation Society - Indonesia Program, Bogor, West Java, Indonesia; National University of Singapore, SINGAPORE

## Abstract

With the extirpation of tigers from the Indonesian island of Java in the 1980s, the endemic and Critically Endangered Javan leopard is the island’s last remaining large carnivore. Yet despite this, it has received little conservation attention and its population status and distribution remains poorly known. Using Maxent modeling, we predicted the locations of suitable leopard landscapes throughout the island of Java based on 228 verified Javan leopard samples and as a function of seven environmental variables. The identified landscapes covered over 1 million hectares, representing less than 9% of the island. Direct evidence of Javan leopard was confirmed from 22 of the 29 identified landscapes and included all national parks, which our analysis revealed as the single most important land type. Our study also emphasized the importance of maintaining connectivity between protected areas and human-modified landscapes because adjacent production forests and secondary forests were found to provide vital extensions for several Javan leopard subpopulations. Our predictive map greatly improves those previously produced by the Government of Indonesia’s Javan Leopard Action Plan and the IUCN global leopard distribution assessment. It shares only a 32% overlap with the IUCN range predictions, adds six new priority landscapes, all with confirmed presence of Javan leopard, and reveals an island-wide leopard population that occurs in several highly fragmented landscapes, which are far more isolated than previously thought. Our study provides reliable information on where conservation efforts must be prioritized both inside and outside of the protected area network to safeguard Java’s last remaining large carnivore.

## Introduction

Carnivores are one of the most threatened groups of terrestrial mammals on earth [1,2]. Within this order, members of Felidae, have suffered the severest population declines and geographical range contractions [1,3,4]. The threats facing large felid species are widely shared. They include habitat loss and fragmentation caused by the expansion of small holder farmland, large-scale monoculture plantations and infrastructure, and also the killing of prey and the felid species itself, in retaliation to conflict or for trade [2,5].

The Endangered leopard (*Panthera pardus*) [6] has the widest geographic distribution of the wild felids [4,7]. It occurs in a broad range of habitats and continents, such as the savannah plains in Africa [4], temperate forests in Russia and China, and humid evergreen rainforests in Southeast Asia [4,8–10]. The species’ behavioral plasticity allows it to survive in areas where other big cats have been extirpated or isolated, such as those close to major human settlements or with an unnaturally low prey base [11–14]. Their high adaptability, however, does not make them tolerant of all threats and a recent assessment revealed that the leopard has lost between 63% and 75% of its historical range, for which a disproportionately high loss (83–87%) has occurred in Asia [4].

The Javan leopard *P*. *p*. *melas* (Cuvier 1809) is one of the most threatened subspecies of leopard. It is endemic to the Indonesian island of Java, which contains 141 million people and has one of the highest human population densities (1,115 people/km^2^) in the world, which greatly restricts the Javan leopard’s island-wide distribution [15]. From the nine recognized subspecies, the Javan leopard is among the three Critically Endangered, along with the Amur leopard and Arabian leopard [16].

Javan leopard is listed on CITES Appendix I [17] and a nationally protected species under Indonesian Government Regulation No. 7/1999. Yet to date, only a few scientific studies have been conducted on the Javan leopard [18–21] and there is a lack of reliable information on its population status across the island.

In 1990, a study recorded that Javan leopard occurred in 12 conservation areas, including national parks, nature reserves, game reserves and hunting reserves, and these supported an estimated population of 350–700 leopards [10]. Later studies, however, found that this subspecies also occurred outside of the conservation area network, such as in production forest and protected forest [22]. In 2013, this led a group of Indonesian scientists to estimated that there were 491–596 leopards in the remaining natural forests across Java [22]. The majority of these leopards existed in ten national parks, of which only two of them, Halimun Salak and Ujung Kulon, were considered to be able to support more than 100 individuals.

A recent global study on leopards found that the Javan leopard occurred in only 16% (20,600 km^2^) of its historical range that had once covered Java [4]. It also found that only 3% of the Javan leopard’s core area now remains and this yielded the lowest median patch size and core area index for any of the leopard subspecies; indicating that it is at a greater risk of extinction than any of the other leopard subspecies. Such spatial extent is much larger than that documented in the Javan Leopard National Action Plan (3% or 3,277 km^2^) [22]. This difference is most likely an artifact of the different spatial datasets used for deriving both estimates. The first estimate was based on 41 records, and restricted to protected areas, while the later on 34 localities with an unclear definition of whether a locality represented a distinct patch or not. This strongly suggests that a more comprehensive and finer-scale estimate is needed to produce a reliable baseline on Javan leopard distribution.

Species distribution modelling based on presence-only datasets is widely used to assist in the management of understudied and threatened species [23–25]. So, in this study we aim to: 1) collate all recent data from 2008–2014 on Javan leopard occurrence; 2) model suitable habitat of Javan leopard, 3) define the extent of suitable habitat inside and outside of the protected area network, 4) investigate land classes that have the potential to support Javan leopard outside of this network, and 5) identify priority actions for future Javan leopard conservation based on our key findings. The main purpose of this study is to provide decision makers and conservation planners alike with the first robust estimate of Javan leopard distribution using a species distribution modelling approach that is based on the most extensive Javan leopard occurrence dataset available.

## Methods

Permits of several projects in this study were obtained from the national parks and the local natural resources conservation agencies, the Indonesian Ministry of Environment and Forestry.

### Data preparation

We collated unpublished Javan leopard points of occurrences recorded by various field research projects and individuals between 2008 and 2014 (S1 Table). For security reasons, we present the approximate location of each Javan leopard locality within a 100 km^2^ rectangle (S1 Fig). We then selected points that had verifiable evidence, including camera trap videos and photographs, human-leopard conflict reports, pugmarks, scratches and feces. Given the absence of other large cats in Java, we believed that misidentification of these signs to be unlikely.

We used ten environmental variables that have been shown to be suitable predictors of large carnivore presence by other studies. These included distance from roads [23], distance from forest edges to the interior and exterior, and protected areas [26], elevation [23,26], distance from rivers [27], slope [23,28], land use classification, precipitation, and temperature [29]. The original spatial dataset included protected area land use classification layers [30], roads, rivers and Indonesia baseline maps [31], digital elevation map (30 m resolution) [32], and mean precipitation and temperature (1 km resolution) [33]. We converted the protected area layer into a binary raster containing '0' (non-protected area) and '1' (protected area).

The original land use classification layer contained 18 classes. For better interpretation, we reduced it to 15 classes based on similarities and converted them into raster layers. We generated the slope layer from the digital elevation map. We selected forest polygons from the land use classification and generated Euclidian distances to produce the distance from forest edge to the Javan leopard points recorded both inside and outside of the primary forest polygons. Thus, for distance from forest edge to the exterior, all Javan leopard points that fell within the forest were assigned a value of '0' and vice versa for the distance from forest edge to the interior. Similarly, we employed Euclidian distance to roads (vector) and rivers (vector) to generate the distance from roads and rivers (30 m resolution) layers, respectively.

The primary analysis tool used in this study required all datasets to have exactly overlapping cells and spatial extent [34], which in this study was confined to the island of Java. We generated a raster mask of 0.25 km^2^ cell size covering Java to provide a baseline environment setting for further resample processing of the background layers. We then re-sampled the Javan leopard points and all environmental variables with the application of a mask layer. This procedure removed any duplication of Javan leopard points within a cell for subsequent analysis. Because several environmental variables were re-sampled from finer to coarser resolution, we performed a bilinear interpolation technique to assign a new value to a cell by using a weighted distance average of four adjacent input cells. All spatial processes were performed using Raster Processing and Spatial Analyst tools in the software ArcGIS 10.2 (ESRI, Redlands).

The Javan leopard points were found to be biased toward the sampled areas, a situation common to presence-only datasets [35]. To control for this sampling bias, we converted the mask layer into a bias grid [36]. In the analysis, the value of a cell (*c1*) in the bias grid was used to assign greater weight to Javan leopard points (*c2*) with fewer spatial neighbors and vice versa. The value of *c1* was a sum of the distances between *c1* and *c2* as calculated using the Gaussian Kernel function, *w* = exp(−*d*^2^/2*s*^2^), where *w* is the weight, *d* is the distance (in km) between *c1* and *c2*, and *s* is the standard deviation. We used 4.2 km for *s* because it represents the known diameter of the largest Javan leopard home range size (13.6 km^2^) [18,19]. Following the Gaussian distribution, *s*, yielded points that are, for example, located 4.2 and 8.4 km away from a cell as having 60.6% and 13.5% as strong an influence, respectively. We used the Distance Among Points tool of the Geospatial Modeling Environment version 0.7.3.0 [37] and the Calculate Variable and Aggregate tools of the IBM SPSS Statistics software version 20 (IBM Corporation 2011) to calculate the distance between *c1* and *c2* and the Gaussian Kernel function, respectively.

### Data analysis

We predicted suitable landscape of Javan leopard using Maxent version 3.4.1. Maxent has been widely used for presence only dataset over other techniques due to its robustness against autocorrelated environmental predictors [38,39], lower sensitivity to small sample sizes [40], and being less affected by spatial errors [41]. The final sample inputs consisted of 169 Javan leopard presence points. We performed a Pearson’s correlation analysis using the Hmsc package within R software (R Development Core Team 2010) to test for correlations between the ten environmental variables, from which a pair of variables was removed if the coefficient correlation was > 0.50 [42]. The final set of uncorrelated environmental variables used in the subsequent analysis included distance from forest edges to the interior and exterior, distance from roads, protected areas, elevation, distance from rivers, land classification and mean precipitation.

We set the protected areas and land classifications as categorical variables and the rest as continuous variables. We performed a Bootstrap procedure with 25% random tests, ten replicates, and 5,000 iterations, and kept the other settings at the default option. We also performed Jackknife tests to assess consistency in variable importance between the training and test gains [43]. The overall model performance was measured by the area under the curve (AUC) of the receiver operating characteristic (ROC) curve [39]. We estimated the relative importance of each predictor to the Maxent model using the percent contribution and permutation importance, averaged over ten replicates. We investigated the response curves to explore how the environmental predictors effected the Maxent prediction.

### Identifying landscapes and defining its characteristics

We used the model prediction to determine suitable Javan leopard landscapes based on a ten percentile training presence logistic threshold. Model pixels with logistic probabilities smaller than the threshold were omitted from the final predictive model [24,34,44]. We converted the predicted suitable patch raster into a polygon format and defined the minimum suitable patch size for Javan leopard as being large enough to contain at least five mature individuals [28]. Subsequently, we retained all predicted suitable patches of at least 68 km^2^ or equal to five times the largest known Javan leopard home range [18,19]. For a Critically Endangered subspecies like Javan leopard, smaller suitable patches with confirmed evidence of Javan leopard may still be important if it supports connectivity between patches. We therefore retained all smaller suitable patches with and/or close to Javan leopard localities up to 4.2 km away or equal to the diameter of their home range. Predicted suitable patches that did not meet these criteria were removed from further analyses.

We assumed all protected areas that fully or partially overlapped with the predicted suitable patches as being important for Javan leopard. We updated these patches with protected areas using the Update tool of the ArcGIS 10.2. This step removed all stand-alone protected areas and combined them with associated suitable patches into one unique polygon. We calculated the area of the identified suitable patches in each protected area as a surrogate indicator of the ecological importance of each protected area to support Javan leopard.

We included the protected area layer in the land use classification layer, so that it would be considered in the analysis. This step removed all land classes that fell within a protected area into one unique polygon coded as “protected area” and produced 16 land use classes. We defined suitable landscapes as patches that: 1) are predicted to be suitable for Javan leopard based on a ten percentile training presence logistic threshold, 2) are larger than 68 km^2^, and/or 3) have evidence of Javan leopard presence, and/or, 4) within 4.2 km of a Javan leopard locality. We then extracted the land use classes in each suitable landscape by intersecting the landscape layer with a land use classification layer using the ArcGIS 10.2 Intersect tool.

## Results

We obtained 228 verified Javan leopard presence points, the majority of which came from a combined record of Javan leopard signs (n = 196) and conflict incidents (n = 32). The elevation range of these data points was 1 to 2,540 m asl, with a mean value of 714 m asl. Approximately half (50.9%) of the leopard records occurred inside the protected area network, with evidence of leopard from all nine national parks in Java. For those records occurring outside of the protected area network (51.6%), most (36.6%) points were located in secondary forest, followed by mixed agriculture (22.3%), production forest (20.5%) and other land use types (20.5%). For the conflict records, most (53.1%) leopard records were recorded from mixed agriculture areas (Table 1).

**Table 1:** Contribution of Javan leopard occurrence records in different land use types. Javan leopard occurrence was identified based on direct and indirect signs, and conflict incidents.

Land use	Conflict	Signs	Total	Percent
Protected area	3	113	116	50.9%
Secondary forest	1	40	41	18.0%
Production forest	3	20	23	10.1%
Mixed agriculture	17	8	25	11.0%
Plantation	2	9	11	4.8%
Rice field	3	4	7	3.1%
Settlement	3	0	3	1.3%
Shrub	0	2	2	0.9%
**Total**	32	196	228	100%

The Maxent analysis identified distance from forest edge and mean precipitation as providing the highest contribution to the predicted leopard distribution, respectively explaining 49.7% and 25.4% of the variation in the predicted suitable patches (Table 2). The Jackknife test for variable importance using the regularized training gain, test gain, and AUC on test data indicated that both distance from forest edge and mean precipitation were the two predictors with the highest gains when used in isolation, which decreased most when these variables were omitted from other candidate models.

**Table 2:** The relative contribution and permutation importance of each variable calculated by Maxent. Values are averaged over the 10 replicates and normalized to give percentages. The permutation importance was used to assess variable importance.

Variable	Percent contribution	Permutation importance
Distance from forest edge to the exterior	49.7	60.1
Precipitation	25.4	21.5
Protected Area	9.8	3.6
Land use type	5.9	4.5
Distance from river	4.1	3.4
Distance from forest edge to the interior	2.9	3.3
Elevation	1.1	1.9
Distance from road	1	1.7

**Fig 1:**
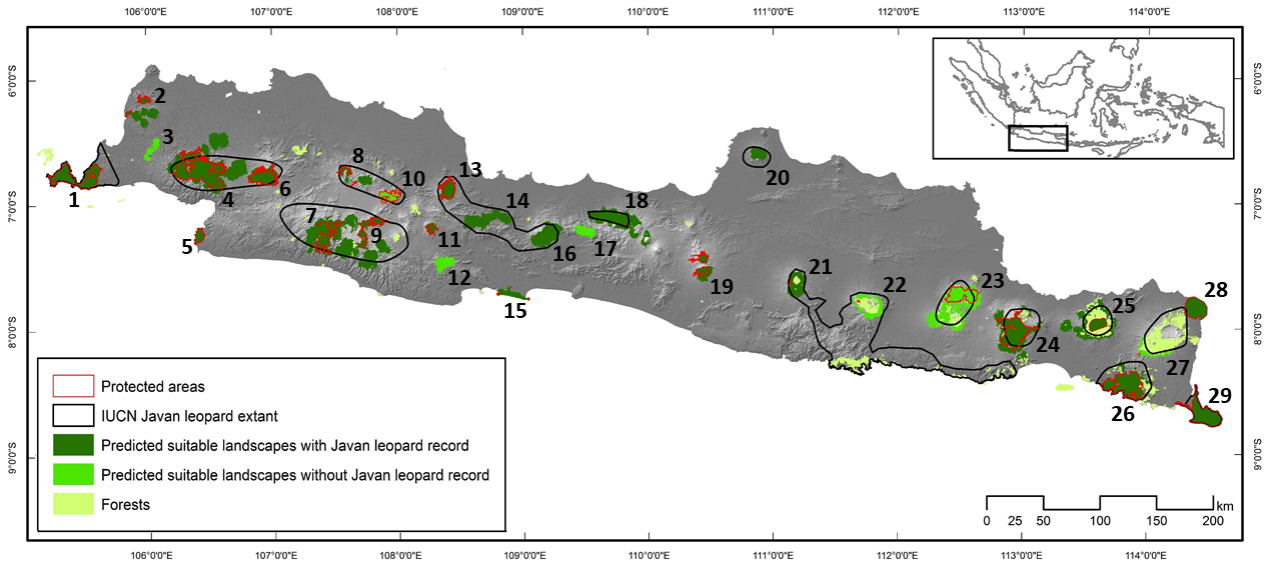
Predictive map identifying suitable Javan leopard landscapes on the Indonesian island of Java. The Maxent model outputs were defined to be suitable for Javan leopard if they had a logistic probability of 0.42 or greater. The numbers represent the 29 predicted suitable landscapes listed in S2 Table.

The response curves indicated that Javan leopard was more likely to occur in forest and in landscapes with higher precipitation (S2 Fig). The species distribution model performed well, with a mean AUC±SE of 0.95±0.007. Applying a ten percentile threshold, only model pixels with a logistic probability of 0.42 or greater were classified as being suitable for Javan leopard. The area of suitable landscape identified was 1,159,864 ha and covered 8.9% of the island of Java [15]. Protected area (40.5%), production forest (17.2%), and secondary forest (13.6%) contributed the largest area (62%) of suitable landscape (S2 Table). Evidence of Javan leopard was recorded from 22 of the 29 suitable landscapes, meaning that seven landscapes would require further field checks to confirm leopard presence (Fig 1).

## Discussion

**Fig 2:**
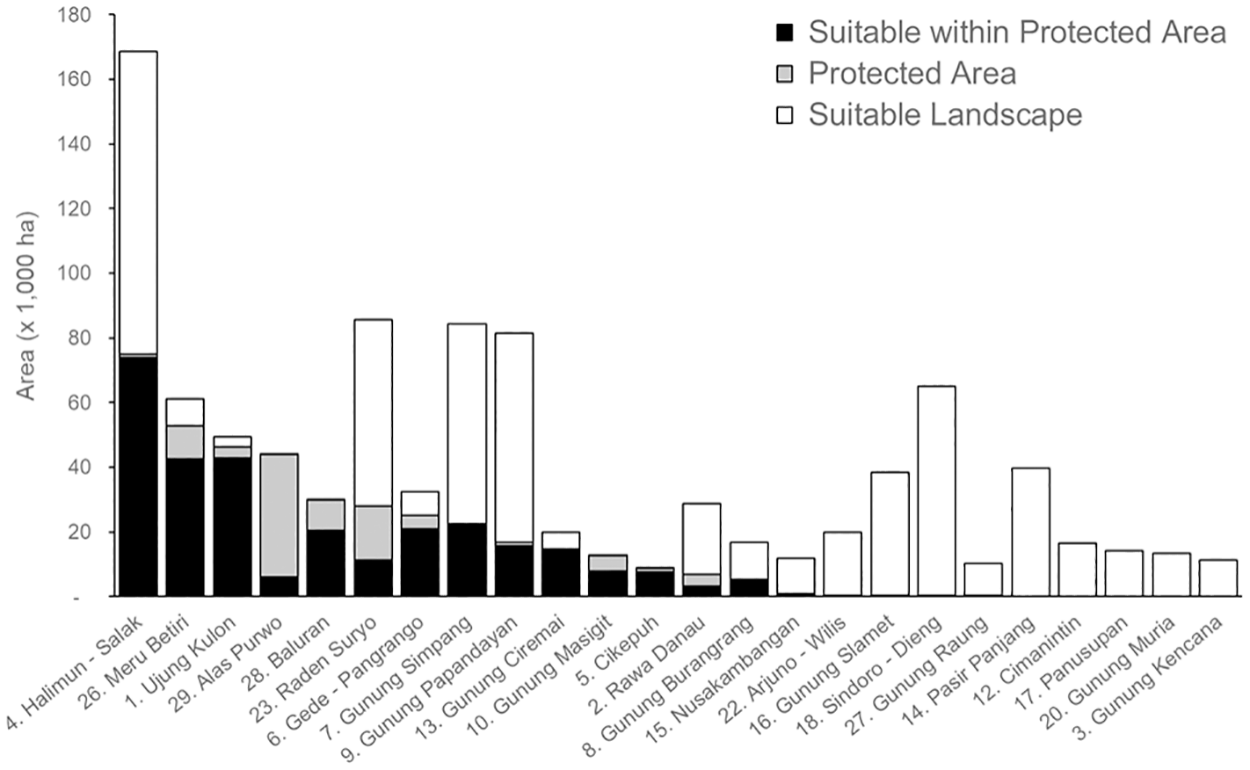
Protected areas in 24 out of 29 suitable landscapes.

The predictive map produced from our study not only refines maps produced from the Government of Indonesia’s Javan Leopard Action Plan [22] and the IUCN global leopard distribution assessment [4,16], but represents the first to be developed from a spatially-explicit modelling process for this subspecies. Our dataset, which is based on 228 occurrence records, is a marked improvement on the 2017 IUCN Javan leopard assessment that was based on significantly fewer (34) data points [16]. Our map shared only a 32% overlap with the IUCN range prediction, but adds six new landscapes (ID: 2, 5, 11, 15, 19, 28), all with confirmed evidence of Javan leopard, and reveals an island-wide leopard population that occurs in several highly fragmented landscapes, indicating that it is far more isolated than previously thought (Fig 2).

### Study limitations

A limitation of this study was that it was unable to incorporate a data layer on prey availability due to the absence of such data from the island of Java [22]. It was not, therefore, possible to investigate the potential of the predicted suitable landscapes, especially those are outside the protected areas and forest habitats, to support viable populations of Javan leopard over the long-term. Future studies should therefore aim to address this question, especially in the priority landscapes. The Javan leopard occurrence data used in our analysis might have certain limitations that are associated with the use of a presence-only dataset. First, data were collated from multiple sources and not based on surveys that used the same sampling method. However, because such a dataset does not exist, we therefore aimed to collect as much data as possible and then filter these to remove redundancy whilst maintaining a comprehensive dataset. Second, although Maxent is less sensitive to small sample sizes [40] and less affected by spatial errors [41], it requires that the samples (occurrence data points) be unbiased and therefore independent of the distribution of the target species [43]. The high AUC value recorded in our model might be an artifact of the AUC statistic, which tends to be higher for species with small home range sizes relative to the study area [43]. However, the potential sampling bias here might have been overcome by our study using a relatively large dataset recorded from all possible habitat types across Java. Furthermore, we controlled for potential sampling bias by using bias grids as a function of their Gaussian Kernel distribution and overcame the dependency issue by resampling the original Javan leopard records into a density of one point per 0.25 km^2^.

### Characterizing Javan leopard landscapes

Our study provides fresh thinking into the role of modified landscapes surrounding protected areas for improving the carrying capacity and, ultimately, long-term survival of the Javan leopard. The Maxent model shows higher association between leopards and more productive landscapes in western Java than the drier forests in eastern Java. This was revealed by the sharp increase in the probability of Javan leopard occurrence in landscapes with higher precipitation. Thus, higher precipitation should support higher plant productivity for the ungulate prey base [45]. However, with the absence of prey data, we were not able to further evaluate the relationship between precipitation and prey base availability. The low contribution of the protected area variable to the overall model output might be explained by the fact that most protected area boundaries were located within larger forest areas, therefore outperformed by the distance from the forest habitats variable.

We identified 70% of the protected area network in Java as providing suitable leopard habitat. However, this network contributed only 40% of the total area of the suitable landscapes. A population viability analysis on ten leopard populations in South Africa predicted that no population with fewer than 50 individuals would survive over 100 years without dispersal and connectivity to a neighboring population [46]. Thus, based on a maximum estimated Javan leopard density, 16 leopards/100 km^2^ [19], 12 suitable landscape patches (#1, 4, 6, 7, 9, 14, 16, 18, 23, 24, 25, 26) were considered large enough to support at least 50 individuals. Here, Javan leopard recovery efforts should focus on strengthening protected area management to reduce poaching and maintain landscape integrity, before moving on to options such as connecting landscapes and their respective leopard populations.

From the landscapes identified with good recovery potential, it is important to stress that, besides the national parks, all other protected area types were found to be too small on their own to hold viable populations of Javan leopard. This situation was also found in several studies in West Africa and North America where many wildlife reserves were to be too small or had inadequate natural resources for achieving large carnivore conservation objectives [47,48]. For the Javan leopard, this highlights the importance of managing suitable landscapes that adjoin these protected areas so as to support viable populations. However, these buffer zone habitats may represent poorer quality habitat and have a lower prey base and therefore support a lower density and number of leopards. Determining leopard densities in these areas and, therefore, how large these areas should be is a topic for future research.

National Park is listed as a Category II protected area of the World Conservation Union [49] and is the strongest protected area type afforded by the Government of Indonesia, in terms of infrastructure, management, financial support and human resources allocated. Thus, strengthening national park management should be considered as a top priority for the long-term survival of the Javan leopard. We identified only three national parks with sufficient habitat to potentially support more than 50 individuals: Gunung Halimun Salak (4, 74,018 ha); Ujung Kulon (1, 42,910 ha); and, Meru Betiri (#26, 42,465 ha). Among these, only Gunung Halimun Salak could potentially support more than 100 individuals, which underlines its importance as a flagship protected area. Two other national parks, Gunung Gede Pangrango (#6) and Bromo Tengger Semeru (#24) could potentially support 50 leopards or more if suitable habitat in the adjacent areas is included.

Understanding the wider landscape characteristics is vital to increasing the population size of large carnivores that are threatened with extinction [50,51]. This not only includes the identification of suitable landscape adjacent to the protected areas, but the key stakeholder groups who should be engaged by conservation managers. Our study found that more than half of the suitable landscape outside of the protected areas is in production forest and secondary forest. Further, with less than 5% of primary forest occurring outside of protected areas, production and secondary forests should therefore play an important role in providing additional habitat for the remaining Javan leopard populations. Here, Government Regulation No. 72/2010 mandates the management of non-conservation state forest in Java to *Perum Perhutani*, a company under the Ministry of State-owned Enterprises, as the main investor, and the Ministry of Environment and Forestry, as an advisor for its technical and operational activities.

Beside state forest, the Government of Indonesia recognizes the legal right of local communities to manage private forest, which is commonly known as *hutan rakyat* (Community Forest). The role of local communities in managing *hutan rakyat* adjacent to state forest is legalized by *Perum Perhutani* through the establishment of a Community Village Forest Institution (*Lembaga Masyarakat Desa Hutan*). Under this scheme, local communities are permitted to develop agroforestry for commodities, such as teak, acacia, silk trees and mahogany. In 2009, 2.7 million ha of *hutan rakyat* in Java was managed by 690,895 households through agroforestry schemes [52,53]. Most *hutan rakyat* is situated adjacent to state forests and may therefore contribute significant Javan leopard habitat that is located around protected areas.

This study confirms the resilience of leopard outside of its main forest habitat type [54]. We found that nearly half of the Javan leopard data points were recorded outside of protected areas and primary forest, which concurs with other studies that found a high level of leopard adaptability in modified habitats [4,7,11,12,24]. This indicates the ability of leopards to subsist in and move through modified habitat [13], as has been found in India [55], South Africa [56] and Russia [57]. These modified habitat may therefore serve as structural corridors that facilitate dispersal to enable source-sink connectivity between viable and otherwise non-viable leopard populations [46]. Balme et al [58] recorded leopards moving far beyond the productive natural habitats and into areas where they were then killed, either deliberately or accidentally, by people. A successful strategy for conserving a wide-ranging large carnivore will therefore rely on protecting the source population and providing dispersal opportunities with sink populations through maintaining connectivity [24,29,59,60]. This should be conducted with a reduction in leopard and prey offtake from hunting, retaliatory killing and problem animal removal [46]. Further studies on Javan leopard movement and home range size as a function of prey availability and potential threats in different habitat types, especially human-modified ones, will allow conservation managers to better understand the species’ response to available resources and different threats, and thus their survival and reproduction potential [61].

More than a quarter of our Javan leopard occurrences were outside of the protected area network and from conflicts with local communities. For a Critically Endangered subspecies, this is of great concern because of the risk posed by retaliatory killings to conflict incidents can have a disproportionately large impact on small population sizes. These types of attacks are well documented in causing negative local community perceptions and attitudes towards the conservation of large carnivores [62–64]. For Java, the history of the tiger’s extirpation from the island approximately 30 years’ ago provides a salutary lesson for the future of the Javan leopard because ultimately the loss of habitat and ensuing competition with people for space and resources, particularly its ungulate prey base, was to the tiger’s great detriment [65]. The Javan leopard, despite being more adaptable than the tiger, now occupies an island that whilst only accounting for 6.9% of Indonesia’s land mass is home to 60% of its human population. This restricts core forest habitat to volcanic peaks and coastal corners. Despite this, our study identifies that many of the occupied landscapes may still contain viable leopard populations, making their protection a top priority. It also identifies smaller landscapes that should be connected to achieve the same aim. Thus, our spatially-explicit modelling provides time-critical information for implementing the 2016–2026 Javan leopard action plan to better effect. This approach could also be adopted for other Critically Endangered species for which there is sparse data, such as the saola.

## Supporting information

S1 Fig The distribution of Javan leopard localities. For security reasons, Javan leopard localities were approximated in 100 km^2^ rectangles.
(TIF)

S2 Fig The response curves of the probability of Javan leopard presence as a function of environmental variables. The curves show the mean response of the 10 replicates (red) and associated one standard deviation (grey area, error bar for categorical variables).
(DOCX)

S1 Table Contributors of Javan leopard records between 2008 and 2014. (DOCX)

S2 Table List of predicted suitable landscapes and land use characteristics in each landscape. Suitable landscapes were defined based on Maxent model outputs with logistic probabilities of 0.42 or greater.
(PDF)
