# Supplementary figures and images for: Identifying priority conservation landscapes and actions for the Critically Endangered Javan leopard in Indonesia: Conserving the last large carnivore in Java Island
Source: PLoS One. 2018 Jun 27;13(6):e0198369. doi: 10.1371/journal.pone.0198369 (PMC6021038; doi:10.1371/journal.pone.0198369)

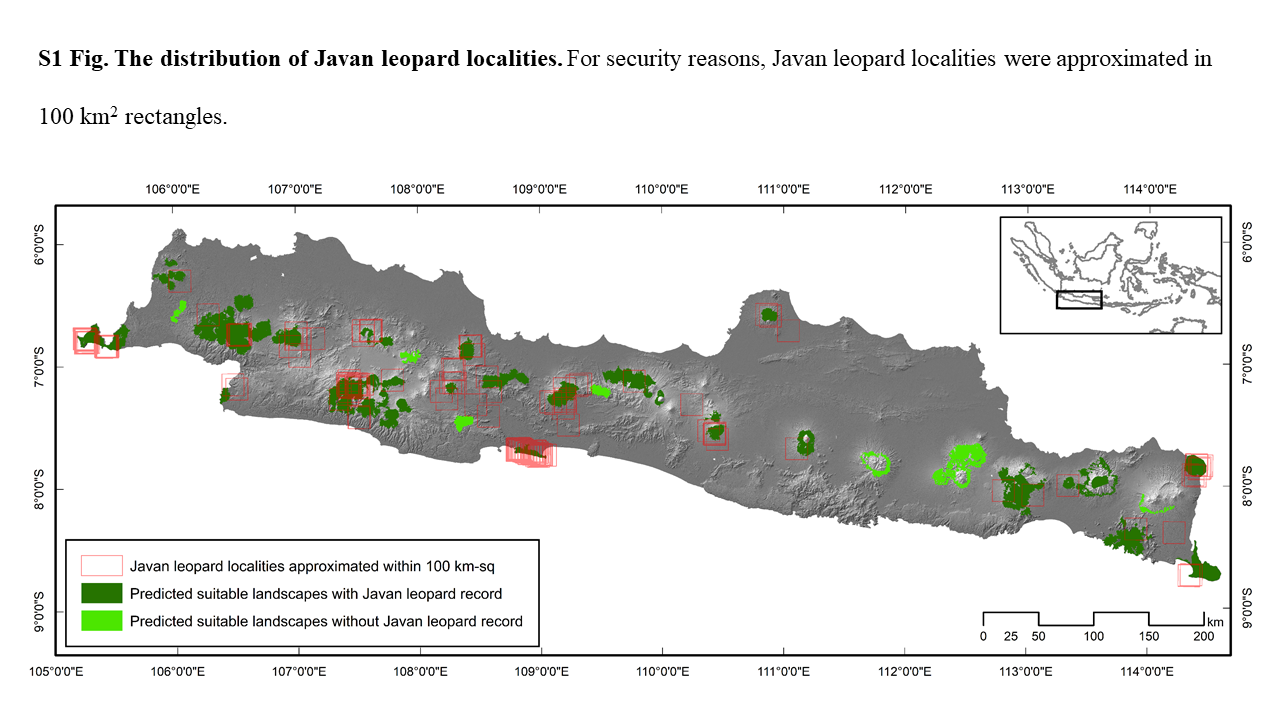

Supplement: S1 Fig — For security reasons, Javan leopard localities were approximated in 100 km2 rectangles. (TIF) [file pone.0198369.s001.tif]
