# Supplementary material for: Identifying priority conservation landscapes and actions for the Critically Endangered Javan leopard in Indonesia: Conserving the last large carnivore in Java Island
Source: PLoS One. 2018 Jun 27;13(6):e0198369. doi: 10.1371/journal.pone.0198369 (PMC6021038; doi:10.1371/journal.pone.0198369)
Supplement: S2 Fig — The curves show the mean response of the 10 replicates (red) and associated one standard deviation (grey area, error bar for categorical variables). (DOCX) [file pone.0198369.s002.docx]

**S2 Fig. Response curves of the probability of Javan leopard presence as a function of environmental variables.** The curves show the mean response of the 10 replicates (red) and associated one standard deviation (grey area, error bar for categorical variables).


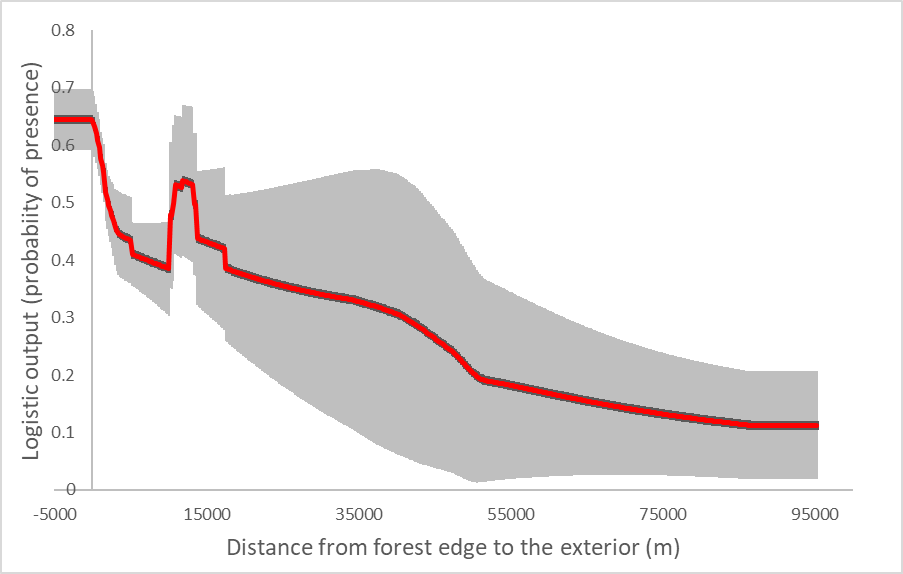

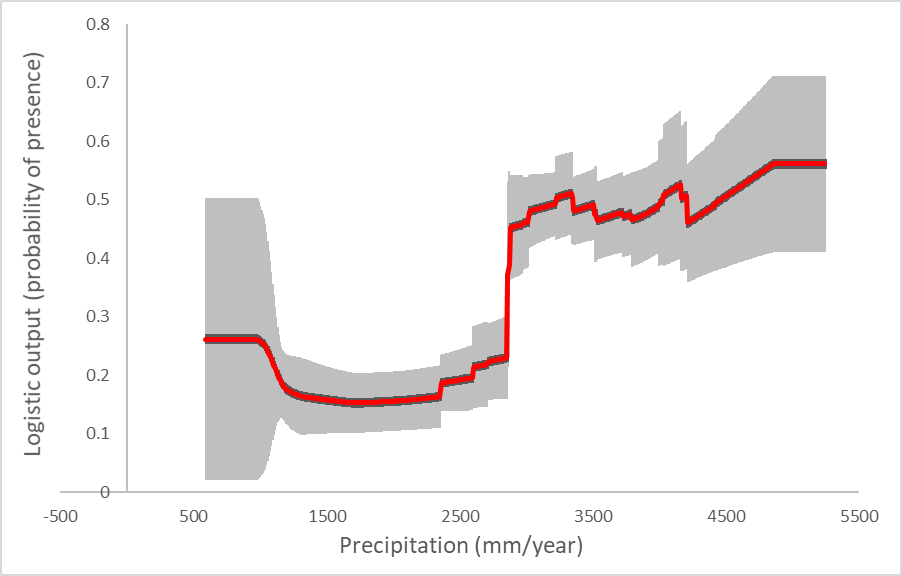


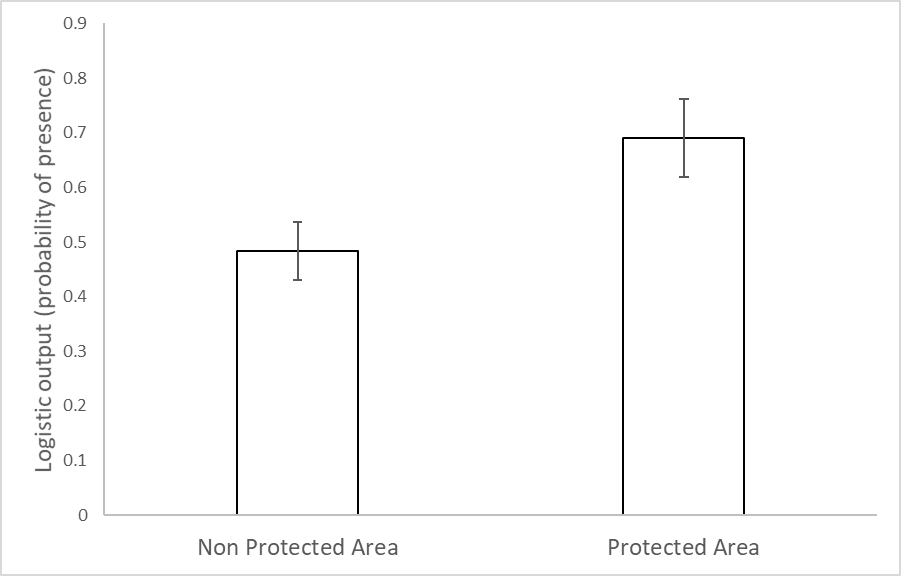

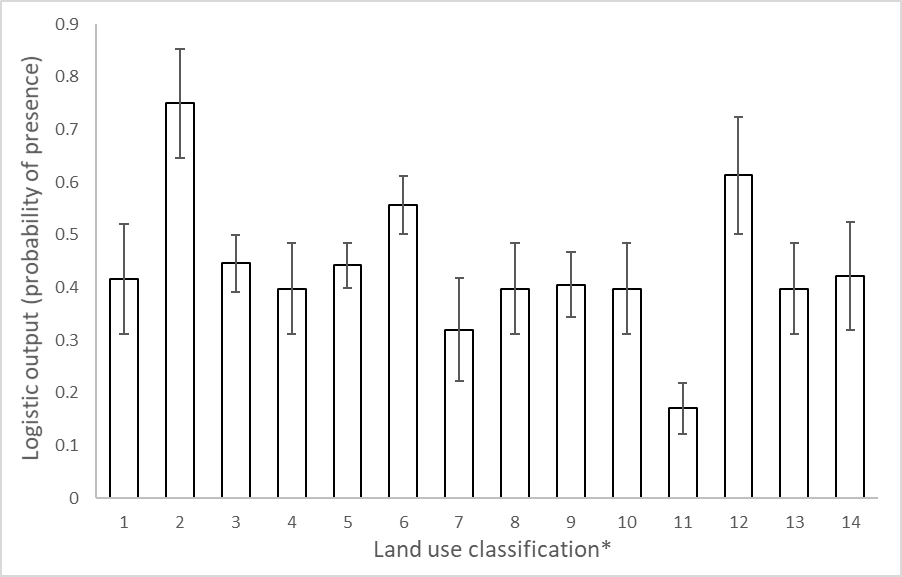


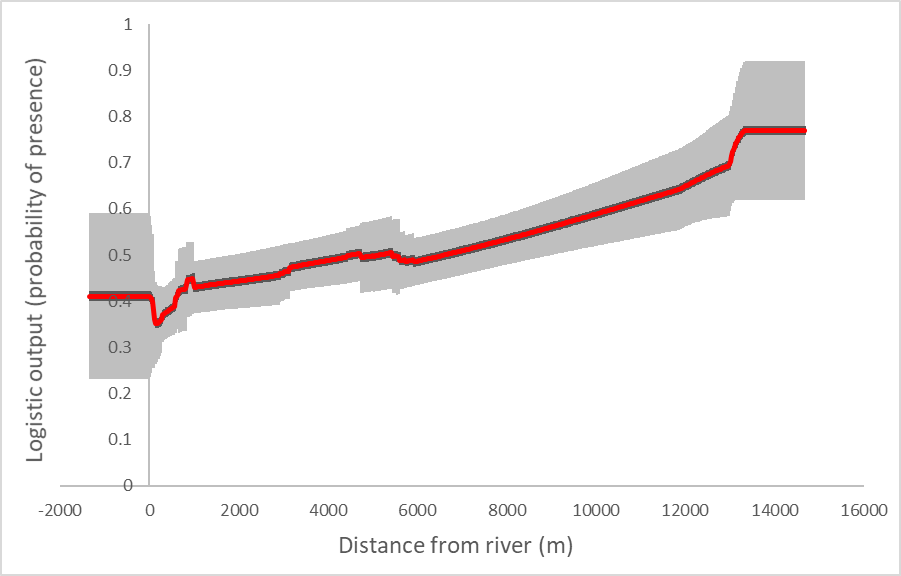

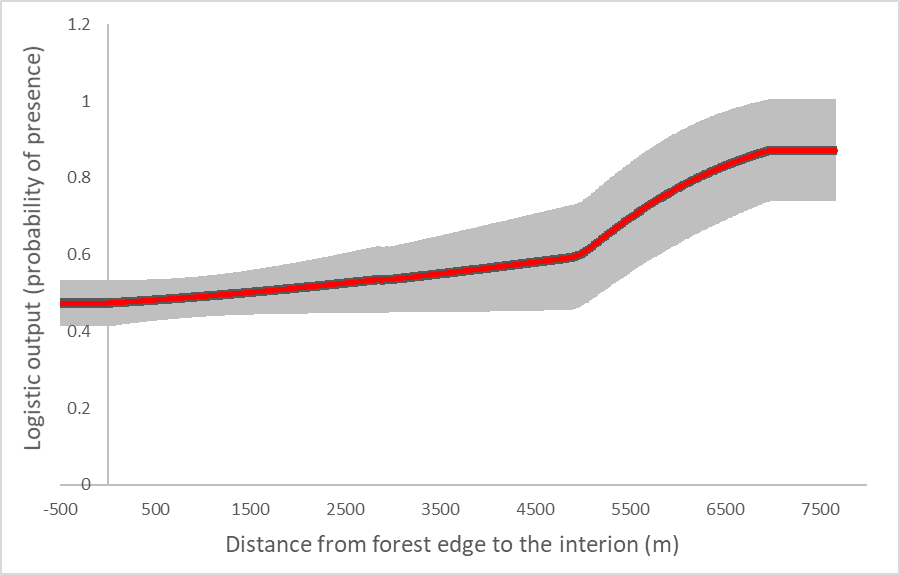


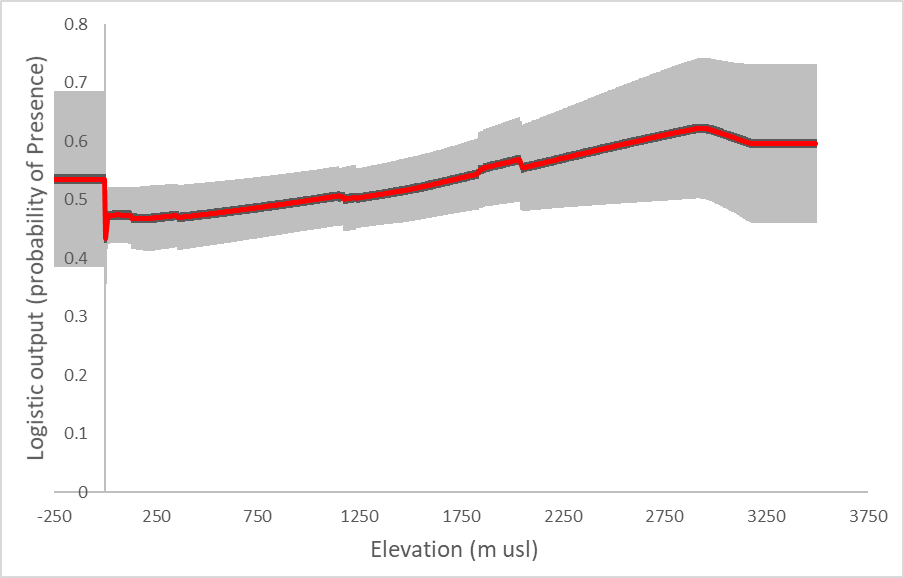

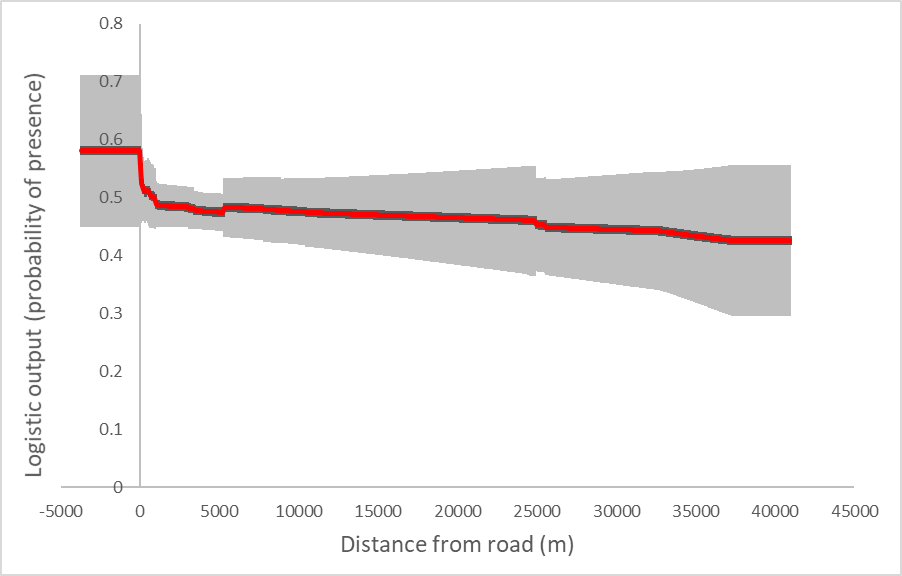


* 1 Primary forest, 2 Secondary forest, 3 Mangrove, 4 Peat forest, 5 Production forest, 6 Plantation, 7 Settlement, 8 Mining, 9 Mixed agriculture, 10 Savanna, 11 Padi field, 12 Shrub, 13 Fisheries, 14 Openland
