# Supplementary material for: Identifying priority conservation landscapes and actions for the Critically Endangered Javan leopard in Indonesia: Conserving the last large carnivore in Java Island
Source: PLoS One. 2018 Jun 27;13(6):e0198369. doi: 10.1371/journal.pone.0198369 (PMC6021038; doi:10.1371/journal.pone.0198369)
Supplement: S1 Table — (DOCX) [file pone.0198369.s003.docx]

**S1 Table.** **Contributors of Javan leopard records between 2008 and 2014.**
