# Supplementary material for: Identifying priority conservation landscapes and actions for the Critically Endangered Javan leopard in Indonesia: Conserving the last large carnivore in Java Island
Source: PLoS One. 2018 Jun 27;13(6):e0198369. doi: 10.1371/journal.pone.0198369 (PMC6021038; doi:10.1371/journal.pone.0198369)
Supplement: S2 Table — Suitable landscapes were defined based on Maxent model outputs with logistic probabilities of 0.42 or greater. (PDF) [file pone.0198369.s004.pdf]

S2 Table. List of predicted suitable landscapes and land use characteristics in each landscape. Suitable landscapes were defined based on Maxent model outputs with logistic probabilities of 0.42 or greater.

Landscape			Protected Area*			Land Use (ha)								
ID	Name	Area (ha)	Name	Status	Area (ha)	Protected Area	Primary Forest	Secondary Forest	Mixed Agriculture	Production Forest	Rice Field	Plantation	Shrub	Others
1	Ujung Kulon	49,332	Ujung Kulon	National Park	46,964	46,241	79	150	1,098	256	1,217	88	107	97
2	Rawa Danau	28,662	Carita	Nature Park	95	6,739	-	3,830	8,405	8,150	1,505	0	16	17
			Rawa Danau	Nature Reserve	3,586									
			Gunung Tukung Gede	Nature Reserve	1,540									
3	Gunung Kencana	11,224				-	-	943	7,940	536	-	566	1,239	-
4	Halimun - Salak	168,723	Gunung Halimun Salak	National Park	75,537	74,923	762	22,746	43,286	10,593	9,740	3,606	2,660	407
5	Cikepuh	8,783	Cibanteng	Nature Reserve	468	8,613	-	-	146	9	14	-	-	1
			Cikepuh	Game Reserve	8,270									
6	Gede - Pangrango	32,370	Gunung Gede Pangrango	National Park	24,614	25,029	59	585	4,958	1,025	-	611	94	8
			Jember	Nature Park	52									
			Telaga Warna	Nature Reserve	296									
7	Gunung Simpang	84,428	Telaga Patengan	Nature Reserve	124	22,421	19	13,803	13,061	8,381	7,931	16,187	2,393	233
			Cimanggu	Nature Park	159									
			Gunung Simpang	Nature Reserve	14,983									
			Gunung Tilu	Nature Reserve	7,574									
			Cigenteng Cipanyi	Nature Reserve	41									
			Malabar	Nature Reserve	29									
8	Gunung Burangrang	16,790	Gunung Burangrang	Nature Reserve	3,270	5,287	-	2,535	2,313	6,189	48	389	29	-
			Gunung Tangkuban Perahu	Nature Reserve	1,220									
			Tahura Ir. H. Juanda	Forest Park	511									
9	Gunung Papandayan	81,422	Gunung Papandayan	Nature Reserve	7,720	16,755	461	23,870	19,993	12,965	4,516	1,190	917	754
			Kawah Kamojang	Nature Reserve	8,236									
			Gunung Guntur	Nature Park	268									
10	Gunung Masigit	12,852	Masigit Kareumbi	Hunting Park	12,613	12,455	24	74	185	98	-	2	13	-
11	Gunung Sawal	7,266	Gunung Sawal	Game Reserve	5,648	5,330	21	-	650	1,198	17	-	50	-
12	Cimanintin	16,383				-	-	3,821	10,051	1,755	546	0	210	-
13	Gunung Ciremai	19,782	Gunung Ciremai	National Park	15,078	14,658	-	25	3,804	811	412	-	72	0
14	Pasir Panjang	39,644				-	-	14,510	14,687	9,468	458	44	461	16
15	Nusakambangan	11,771	Nusakambangan Barat	Nature Reserve	667	852	-	6,772	2,041	-	-	-	286	1,821
			Nusakambangan Timur	Nature Reserve	214									
			Karang Bolong	Nature Reserve	<1									
16	Gunung Slamet	38,326	Telaga Ranjeng	Nature Reserve	57	54	84	10,341	13,946	12,458	456	-	584	404
17	Panusupan	14,122				-	-	1,259	4,899	7,916	50	-	-	-
18	Sindoro - Dieng	64,935	Telogo Dringo	Nature Reserve	29	31	-	16,447	9,659	37,608	904	120	166	-
19	Merapi - Merbabu	19,488	Gunung Merbabu	National Park	5,924	12,293	-	241	2,646	4,028	126	-	-	154
			Gunung Merapi	National Park	6,728									
20	Gunung Muria	13,236				-	-	3,770	3,653	5,814	-	-	-	-

Landscape			Protected Area*			Land Use (ha)								
ID	Name	Area (ha)	Name	Status	Area (ha)	Protected Area	Primary Forest	Secondary Forest	Mixed Agriculture	Production Forest	Padi Field	Plantation	Shrub	Others
21	Grojogan Sewu	26,260	Grojogan Sewu	Nature Park	63	311	-	7,457	7,827	9,582	625	300	156	-
			KGPAA Mangkunegoro I - Ngargoyoso	Forest Park	254									
22	Arjuno - Wilis	19,833	Sigogor	Nature Reserve	197	188	-	5,892	4,515	8,786	-	-	309	142
23	Raden Suryo	85,670	Raden Soeryo	Forest Park	28,134	28,017	13,607	3,072	11,572	20,264	3,333	3,244	1,904	657
			Tretes	Nature Park	19									
24	Bromo - Tengger - Semeru	87,920	Bromo Tengger Semeru	National Park	50,243	50,396	1,383	7,654	13,022	10,983	2,793	-	1,427	261
25	Dataran Tinggi Yang	55,530	Dataran Tinggi Iyang	Game Reserve	13,804	12,865	7,932	4,709	4,301	16,037	2,667	5,285	1,711	23
			Sungai Kolbu Iyang Plateau	Nature Reserve	19									
26	Meru Betiri	61,149	Meru Betiri	National Park	53,758	52,756	-	2,881	7	393	6	2,252	2,856	-
27	Gunung Raung	10,236	Curah Manis Sempolan I - VIII	Nature Reserve	18	17	810	908	76	4,542	-	1,809	1,928	147
28	Baluran	29,847	Baluran	National Park	30,183	29,804	-	4	-	7	30	-	0	0
29	Alas Purwo	43,879	Alas Purwo	National Park	45,004	43,785	8	-	-	1	-	-	66	20
		1,159,864				469,821	25,248	158,299	208,739	199,854	37,393	35,694	19,654	5,161

* Statistik Direktorat Jenderal KSDAE 2015. Jakarta, Indonesia.
